# Research on Pedestrian Detection Model and Compression Technology for UAV Images

**DOI:** 10.3390/s22239171

**Published:** 2022-11-25

**Authors:** Xihao Liu, Chengbo Wang, Li Liu

**Affiliations:** 1 Aerospace Information Research Institute, Chinese Academy of Sciences, Beijing 100094, China; 2 University of Chinese Academy of Sciences, Beijing 100049, China

**Keywords:** pedestrian detection, UAV, small target, model compression

## Abstract

The large view angle and complex background of UAV images bring many difficulties to the detection of small pedestrian targets in images, which are easy to be detected incorrectly or missed. In addition, the object detection models based on deep learning are usually complex and the high computational resource consumption limits the application scenarios. For small pedestrian detection in UAV images, this paper proposes an improved YOLOv5 method to improve the detection ability of pedestrians by introducing a new small object feature detection layer in the feature fusion layer, and experiments show that the improved method can improve the average precision by 4.4%, which effectively improves the pedestrian detection effect. To address the problem of high computational resource consumption, the model is compressed using channel pruning technology to reduce the consumption of video memory and computing power in the inference process. Experiments show that the model can be compressed to 11.2 MB and the GFLOPs of the model are reduced by 11.9% compared with that before compression under the condition of constant inference accuracy, which is significant for the deployment and application of the model.

## 1. Introduction

In recent years, thanks to the rise of UAV remote sensing technology and its advantages such as fast response and global view, UAV image pedestrian object detection technology has been playing an important role in emergency search and rescue and law enforcement tracking [1]. However, there are two problems that need to be solved: first, the UAV remote sensing images have large view angles and complex backgrounds, and the pedestrian targets in the images are small in size, so they are easily missed or misidentified; second, the object detection algorithms based on deep learning are often computationally intensive and possess high requirements for hardware computing power, so the application scenarios are limited. However, the structure of high-precision detection models is usually more complex and requires more computational resources, so it is difficult to achieve a balance between efficiency and accuracy to ensure detection accuracy while minimizing computational consumption for a wider range of applications.

Since the time when convolutional neural networks were first applied to object detection tasks, deep learning-based object detection methods have achieved widespread use in industry with powerful feature extraction and adaptive learning capabilities, far outperforming traditional object detection methods in terms of detection performance [2]. In the field of pedestrian object detection, more and more scholars have improved the deep convolutional neural network structure and achieved good detection results. Hui et al. [3] verified the effectiveness of Faster RCNN [4] for pedestrian detection by incorporating K-means clustering algorithm and RPN network to generate suggested candidate regions, and then classified and localized pedestrian targets by detection network. Qian et al. [5] proposed a PVDNeT network by improving the network structure, and both pedestrian and vehicle detection accuracies were significantly improved compared with the original Faster RCNN algorithm.

However, these studies mostly take the industry applications of video surveillance, autonomous driving, and intelligent robotics as the starting point, and use data acquisition methods mainly from near-parallel views such as roadside surveillance cameras and in-vehicle cameras. The pedestrian target size in UAV images is small, while the target size in natural images is usually large, and the corresponding algorithms are difficult to be fully applicable to the pedestrian detection task in UAV images. To address such problems, Liu et al. [6] enriched the feature information by adding convolutional layers to the YOLOv3 network structure, which in turn enhanced the detection capability of small-sized pedestrians. Mao et al. [7] enhanced the information extraction capability of the network in spatial dimensions by applying multi-scale segmentation attention units to deep neural networks, which improved the pedestrian detection in complex backgrounds. Wu et al. [8] improved the average accuracy rate by 5.09% over the original YOLOv4 network by expanding the object detection scale and introducing the attention mechanism. Zhang et al. [9] proposed an improved lightweight network MobileNetv3 based on YOLOv3 to reduce algorithm complexity and constructed a new attention module SESAM in MobileNetv3 to judge long-distance and small-volume objects. Considering the limited computing power of UAV platforms, Li et al. [10] proposed a lightweight combinational neural network ComNet for object detection in UAV-borne thermal images. The experimental results show that the average precisions for pedestrian and vehicle detection improved by 2%∼5% compared with YOLOv3 model. Jin et al. [11] utilized one emerging method based on YOLOv3 in high-density pedestrians detection situations and achieved good results. To improve the near-surface detection performance of UAVs in low illumination environments, Wang et al. [12] proposed a U-type generative adversarial network (GAN) to fuse visible and IR images to generate color fusion images. Then, a YOLOv3 model combined with transfer learning was trained using the fused images and achieved good results. Kong et al. [13] proposed an improved YOLOv4 model for pedestrian detection and counting in UAV images, named YOLO-CC. YOLO-CC replaces the backbone with CSPDarknet-34, and two feature layers are fused by FPN. By embedding the density map generation method into the network, YOLO-CC can make feature extraction more focused on small targets. Ma et al. [14] proposed a small-sized pedestrian detection algorithm based on the weighted fusion of static and dynamic bounding boxes. The experimental results showed that the proposed method was better than the mainstream object detection algorithm. Shao et al. [15] proposed a method of aerial infrared YOLO (AIR-YOLOv3), which combines network pruning and the YOLOv3 method. Compared with the original YOLOv3, AIR-YOLOv3 has smaller model size while the model AP decreased by only 1.7%.

## 2. Related Theories

### 2.1. Single-Stage Object Detection Algorithm

The single-stage object detection algorithm does not require the suggestion frame stage in the two-stage approach and can directly generate the class probability and position coordinate values of the object, i.e., the image can be directly detected after a single detection to obtain the final detection result. The YOLO family of algorithms is a classical single-stage algorithm that has been iteratively improved since the birth of YOLOv1 [16]. Now, YOLO algorithm has been well-applied in many industries. Khasawneh et al. [17] used YOLOv3 to perform automatic K-complex detection in real-time with high accuracy that aid practitioners in speedy EEG inspection. Huang et al. [18] proposed an improved YOLOv3 detection method for immature apples in the orchard scene and provided a feasible solution for the automation and mechanization of the apple industry. Abdusalomov et al. [19] presented a method for real-time high-speed fire detection using YOLOv3 and detected fire candidate areas and achieved a seamless classification performance compared with other conventional fire detection frameworks.

**Figure 1:**
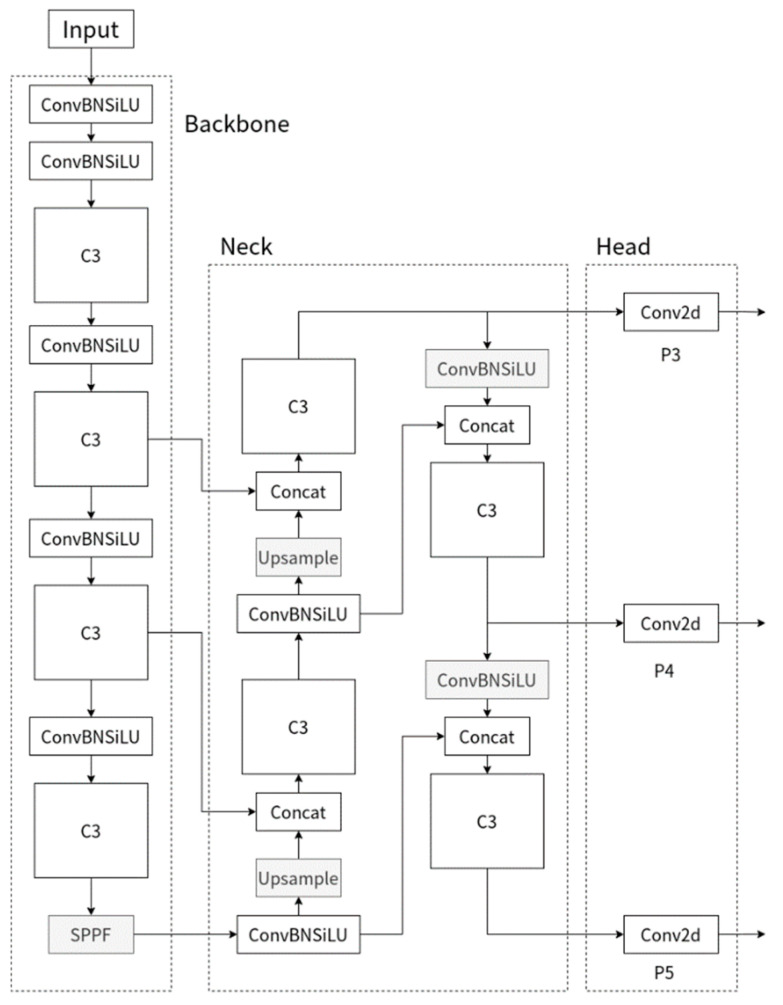
YOLOv5 algorithm structure diagram.

YOLOv5 is one of the widely used object detection networks, which has achieved good results in various industrial problems by virtue of high detection accuracy and fast inference. YOLOv5 is similar to the network structure of YOLO series, Figure 1 shows the YOLOv5 network structure, which consists of an input layer (Input), a backbone feature extraction network (Backbone), a feature fusion layer (Neck), and output layer (Head). Among them, input is a three-channel RGB image with an image size of 640 × 640 × 3, and mosaic data enhancement is used to enrich the detection target image and reduce the model’s dependence on batch size. Backbone is new CSP-Darknet53 which uses BottleNet structure for feature extraction. New CSP-Darknet53 mainly consists of C3 and SPPF structures. The C3 module, by improving the CSP module used in the YOLOv4 [20] model, enhances the ability of model to capture features. The SPPF structure replaces the Spatial Pyramid Pooling (SPP) [21] structure to improve the computational speed of the model. Neck is a structure combining Feature Pyramid Network (FPN) and Path Aggregation Network [22] (PAN), which fuses the semantic information extracted by the deep network with the location information extracted by the shallow network. At the same time, feature fusion is performed between Backbone and Neck to enable the model to obtain more abundant feature information. Head has three detectors to predict the results for different size image features.

Although YOLOv5 has made good achievements, there are certain shortcomings, such as there is room for improvement in multi-scale object detection tasks containing small targets, and it requires high hardware computing power. Therefore, in this paper, YOLOv5 is improved and optimized in terms of algorithm model complexity and detection accuracy.

### 2.2. Model Compression Methods

**Figure 2:**
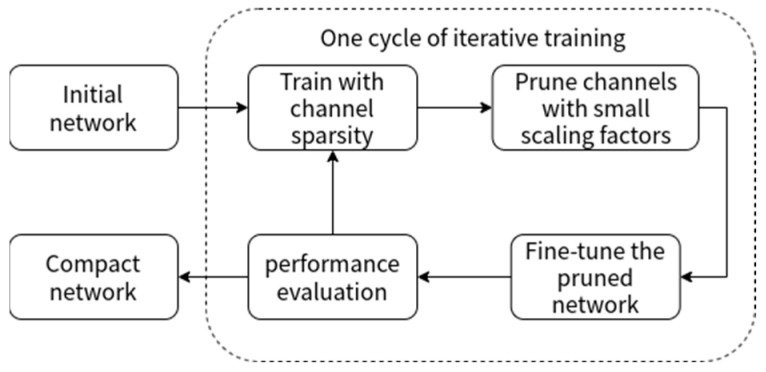
Model compression process.

Model compression methods generally include the main steps of sparse training and channel pruning. The purpose of sparse training is to make the weights of unimportant channels converge to 0, thus preserving important information on a small number of channels [23]. As the weights of most channels converge to 0, the network becomes increasingly sparse, usually by using the Batch Normalization (BN) layer [24], which is used extensively in convolutional neural networks. Channel pruning is used to obtain a lightweight model by setting a suitable threshold for the weight of the model channels, and then cropping out the channels with weights less than the threshold [25], and finally fine-tuning the training so that the accuracy of the pruned model is improved [26]. By iterating the above process until the accuracy of the model meets the application requirements, the process is shown in Figure 2.

## 3. Research Methodology

### 3.1. Improved YOLOv5-Based Pedestrian Detection Algorithm for UAV Images

The original YOLOv5 network uses three different sizes of feature maps to detect targets of different sizes, and three different scales of feature maps are obtained by 8×, 16×, and 32× down-sampling, and their feature map sizes are 80 × 80, 40 × 40, and 20 × 20 when the input image is 640 × 640 size; among them, the 80 × 80 feature map is used to detect small targets, and the 8 × 8 image region corresponds to a pixel on this feature map.

**Figure 3:**
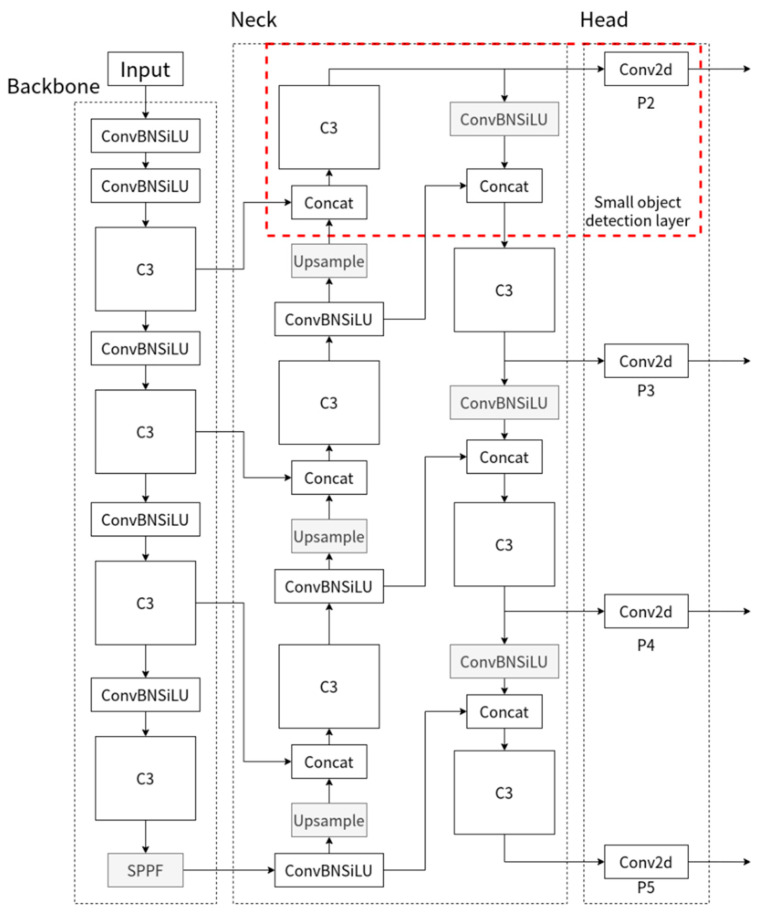
Structure diagram of improved YOLOv5 algorithm.

However, considering that the UAV image size is usually above 1000 × 1000 pixels, the proportion of pedestrian targets is generally small, and the receptive field of 8 × 8 is difficult to express small target pedestrian features. To enhance the detection capability of the network for small target pedestrians without expanding the resolution of the input image, a detection layer for small targets is added. As shown in Figure 3, a channel to Neck is added in the first C3 module of Backbone to fuse with the bottom features after up-sampling to obtain more semantic information, which becomes an independent P2 small-target detection head in Head after a C3 module extracts features for output. In the case that the input image is 640 × 640 size, the feature map size of this detection head is 160 × 160, and each feature image element corresponds to the perceptual field of 4 × 4 of the input image, which facilitates the detection of smaller targets. Meanwhile, this channel in the PAN structure provides more position information to the P3, P4, and P5 detection heads by down-sampling to enhance the overall prediction accuracy of the network.

### 3.2. Model Compression

#### 3.2.1. Sparse Training

In convolutional neural networks, the BN layer can be used to make the network converge quickly and improve the generalization ability of the network. Moreover, in the model compression task, the BN layer can be used to determine the importance of each channel in the information flow by normalizing the ability to process the data of each channel to filter out a small number of important channels and achieve the purpose of network sparse. The formula of BN layer is shown in Equation (1).

(1) $$\hat{z}=\frac{z_{in}-\mu_{ℬ}}{\sqrt{\sigma_{ℬ}^{2}+\epsilon }};z_{out\;}=\gamma \hat{z}+\beta$$

where $z_{in}$, $z_{out\;}$ are the input and output data of the BN layer, respectively; $\mu_{ℬ}$, $\sigma_{ℬ}^{2}$ are the mean and variance calculated from the input data of each network layer, respectively; and γ, β are the scale and offset factors that play a linear transformation in the BN layer, respectively.

From Equation (1), we can see that γ, as the coefficient of the normalized input term, directly affects the proportion of input information in the output result. During the training process of the network, if a channel contains information important to the target classification, its corresponding γ coefficient is stimulated by the loss function and become larger; if a channel contains information irrelevant to the classification, the γ coefficient keeps becoming smaller under the influence of the loss function. Therefore, the γ coefficients converge to a stable value after the training is completed, and this value can be a quantitative indicator of the importance of the channels.

In order to improve the sparsity of the network using the γ coefficient, the network sparsity can be combined with the training process of the neural network to reconstruct the loss function as shown in Equation (2).

(2) $$L=\underset{\left(x,y\right)}{\sum }l\left(f\left(x,W\right),y\right)+\lambda \underset{\gamma \in \Gamma }{\sum }g\left(\gamma \right)$$

where $\left(x,y\right)$ is the training input and target, W is the training weight, λ is the penalty term, and $g\left(\gamma \right)$ is the L1 regularization. The first term in Equation (2) keeps the training loss function of the original CNN unchanged, and the second term is the penalty function $g\left(\gamma \right)$ = |γ| imposed on the scaling factor γ. This penalty function allows the network to further concentrate the weight distribution at the important channels while optimizing the loss function normally.

#### 3.2.2. Channel Pruning

First, the absolute values of the sparse scaling factor γ are sorted in ascending order, while a pruning rate (between 0 and 1) is specified for the whole network according to the demand, which represents the degree of network volume reduction. Next, the corresponding numbers of convolutional channels with smaller γ are pruned according to the pruning rate, and finally a compact network is obtained. Generally, the network performance is reduced after pruning, and in order to recover the network performance, the compressed model needs to continue iterative training to fine-tune the network weights. Algorithm 1 shows the process of model compression.
**Algorithm 1** Process of model compression**Input**: M layers of model, pruning rate α(0<α<1)**Output**: compact model**while** (experimental results meet the requirements) do Sparsity training and get sparse scaling factor $\gamma_{j}^{i}$ of *j*-th channel of *i*-th layer  Sort $\gamma_{j}^{i}$ from small to large and get new list
 *L* Threshold
$\;t=L\left[int\left(\alpha \cdot len\left(L\right)\right)\right]$
  **for**
i=1 to M do  **for**
j=1 to N(channel numbers of *i*-th layer) do    **if**
$\gamma_{j}^{i}<t$ delete *j*-th channel of *i*-th layer  
**end for**
 
**end for**


## 4. Experiments and Results

### 4.1. Experimental Data

**Figure 4:**
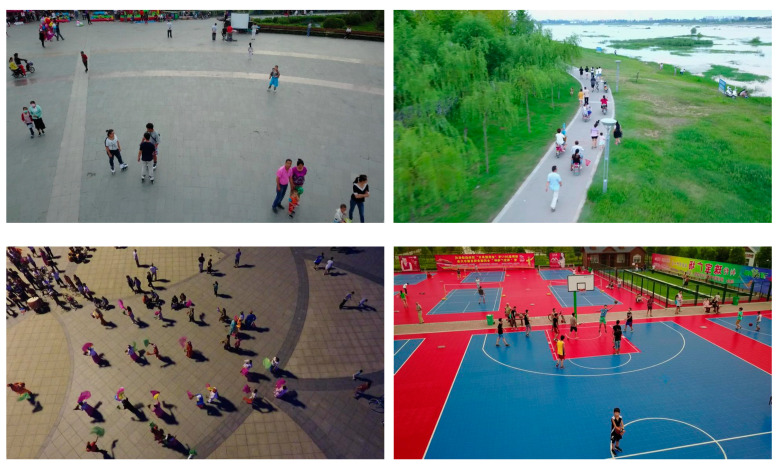
Images of pedestrian feature-rich images in VisDrone dataset.

**Figure 5:**
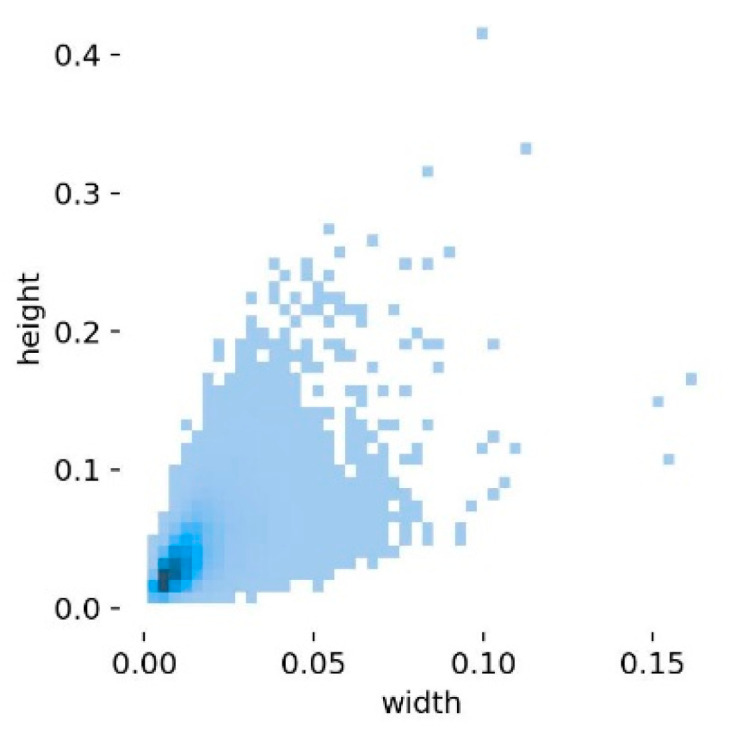
Label size distribution of the training set.

The experimental data for pedestrian detection were obtained from the VisDrone public dataset [27], which was collected and created by the AISKYEYE team at the Machine Learning and Data Mining Laboratory of Tianjin University. The dataset covers different scenarios under neighborhoods and suburbs in 14 cities in China, covering diverse weather and lighting conditions, including people, pedestrians, cars, vans, buses, trucks, motorcycles, and other targets in a total of ten. The dataset consists of 263 videos and 10,209 still images, of which the VisDrone-DET dataset for image object detection is divided into 6471 training sets, 548 validation sets, and 3190 test sets. In this experiment, images with pedestrian annotations (as shown in Figure 4) are selected from typical scenes such as urban, suburban, night, and daytime, and the data with the problem of missing markers are eliminated, and the two types of targets, pedestrians and people, are combined into one category of pedestrians to form an experimental dataset with 4634 pedestrian feature-rich images. The dataset is divided into training, validation, and test sets in the ratio of 7:2:1, which is used in this experiment training, validation, and testing of the pedestrian detection network model in this experiment. Figure 5 shows the distribution of all label sizes in the dataset, the horizontal coordinates represent the ratio of target frame width to image width and the vertical coordinates represent the ratio of target frame height to image height. It can be found that the ratio of pedestrian target size to image size in this dataset is generally small.

Considering that the pedestrian sizes in the VisDrone dataset are generally small, we used the K-means method to re-cluster the anchors in the VisDrone dataset in order to improve the accuracy of the model. As shown in Table 1, the original YOLOv5s model clustered to obtain 9 anchors corresponding to 3 detection layers of different scales, and the improved YOLOv5s_P2 model clustered to obtain 12 anchors corresponding to 4 detection layers of different scales.

### 4.2. Accuracy Metrics

In this paper, we used the accepted performance evaluation metrics in the field of object detection: Precision, Recall, and Average Precision (AP) under different Intersection over Union (IoU) thresholds to measure the detection accuracy of the algorithm [28]. In this paper, the value of the IoU used to produce the results is 0.5.

Precision is the ratio of the number of pedestrians correctly detected by the model to the total number of pedestrian targets identified as pedestrians in the test set; Recall is the ratio of the number of pedestrians correctly detected by the model to the total number of pedestrian samples in the test set; and AP value is a comprehensive evaluation metric determined by the area under the P–R curve plotted by Precision and Recall, the better the algorithm detection effect the higher the detection accuracy. Recall and Precision are defined as:

(3) $$Precision=\frac{TP}{TP+FP}$$

(4) $$Recall=\frac{TP}{TP+FN}$$

where TP denotes the pedestrian samples correctly identified, FP denotes the background samples misidentified as pedestrians, and FN denotes the pedestrian samples misidentified as background.

### 4.3. Model Training

**Figure 6:**
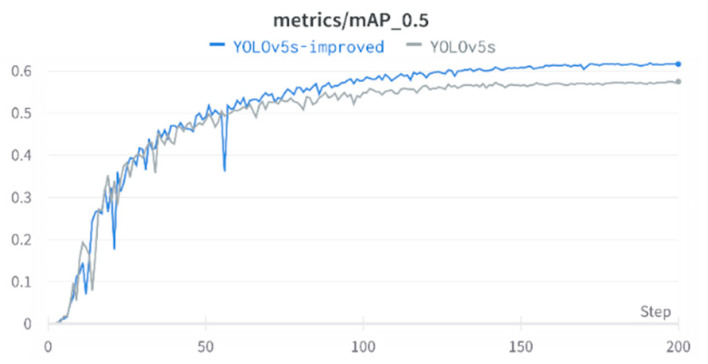
YOLOv5s vs. YOLOv5s-improved method Average Precision comparison on the validation set.

**Figure 7:**
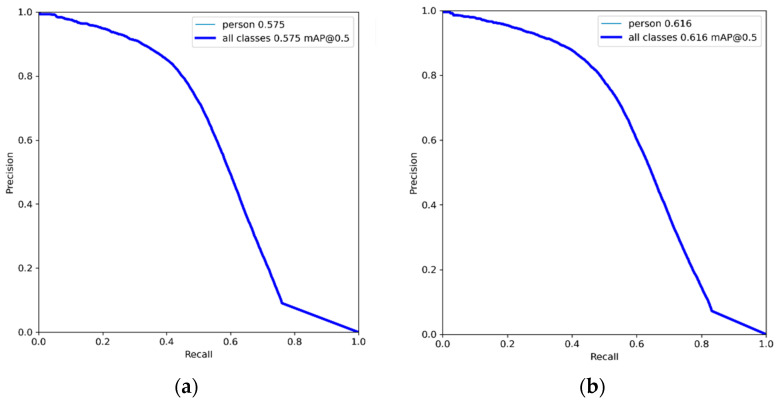
YOLOv5 (**a**) vs. YOLOv5s-improved (**b**) method Precision–Recall curves on the validation set.

In this paper, the YOLOv5s model is selected and trained under Ubuntu 18.04 operating system (Dell Co., Ltd., Beijing, China) with the deep learning framework Pytorch 1.12.0, and the image processor (GPU) is NVIDIA GeForce GTX TITAN X (12GB video memory). Using the default hyperparameters of the YOLOv5s network, the training and test image sizes were set to 640 × 640, the batch size used for the model was set to 16, and the number of training epochs was set to 200. The training process is shown in Figure 6, and the accuracy of the model gradually increases until convergence as the number of epochs increases. In Figure 6, YOLOv5s represents the original model, and YOLOv5s-improved represents the model with the addition of small object detection layer. It can be seen from the figure that the accuracy of the improved model is higher than that of the original model. The Precision–Recall curves of YOLOv5s and YOLOv5s-improved are shown in Figure 7.

**Figure 8:**
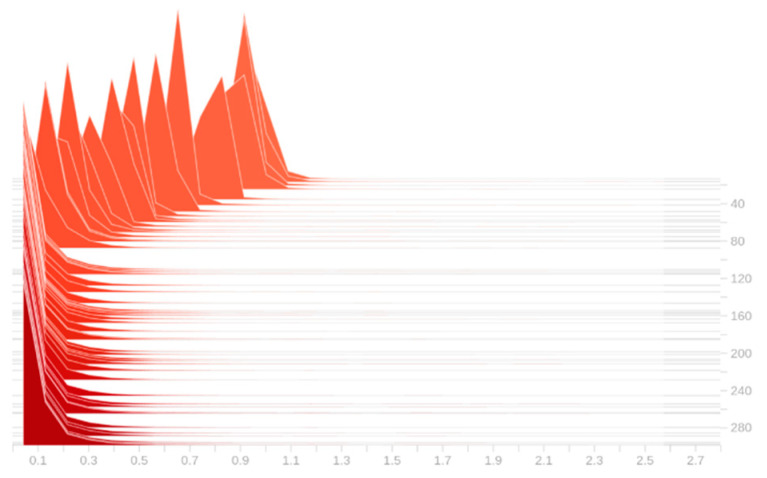
Variation in γ parameters of BN layer during sparse training.

**Figure 9:**
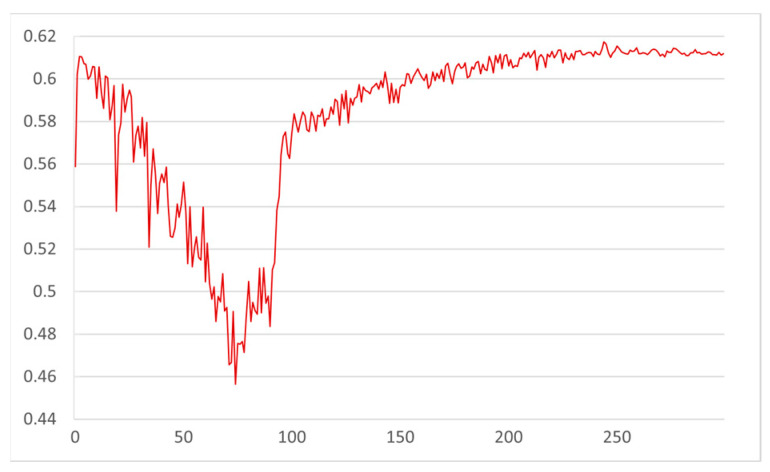
Variation in model accuracy during sparse training.

The pruning coefficient λ is set to 0.005, and the model with the addition of the small object detection layer is trained sparsely. As shown in Figure 8, with the sparse training of the model, the γ parameter of the BN layer gradually converges to 0. After 300 epochs of training, the model converged, and at this time most of the γ parameters of the BN layer converged to 0, indicating that there are indeed low-information channels in the network model, which lays the foundation for the channel pruning later. Figure 9 shows the changes in the model accuracy on the validation set during the sparse training process. It can be seen that the accuracy of the model gradually decreases between 0 and 80 epochs, which corresponds to the process that the γ parameters of the BN layer converge to 0 rapidly in Figure 8; in the subsequent 220 epochs, the distribution of the γ parameters of the BN layer no longer changes significantly, while the the accuracy of the model also gradually rises and returns to the original value until convergence. After the sparse training, the pruning ratio is set to 0.3 for channel pruning, and the pruned model is fine-tuned for training to obtain the final lightweight model.

### 4.4. Experimental Analysis

After improvement and compression, the model was evaluated for accuracy on the test set, and the results are shown in Table 2. Compared with the original YOLOv5s model, the improved model with the addition of the small object detection layer (YOLOv5s_P2) improved 1.3% in accuracy, 3.4% in Recall, and 4.4% in AP. The introduction of the small object detection layer does improve the overall accuracy, especially on the recall rate, indicating that the problem of missing small-sized pedestrians in the original YOLOv5s model was alleviated to some extent. The introduction of the small object detection layer makes the minimum down-sampling of the detection head of YOLOv5 model 4× rather than 8×. When the input size of image is 640 × 640, a pixel of the feature map in the small object detection layer corresponds to the 4 × 4 image area, which matches the size of the small target.

At the same time, the introduction of the small object detection layer also brings additional computational overhead, with the size of the model expanding from 14.4 MB to 15.2 MB, GFLOPs of the model expanding from 15.8 to 18.5, and the single picture detection time rising from 4.6 ms to 7.5 ms. This also shows the limitation of this method: the introduction of small objects detection layer inevitably leads to the increase in model parameters, model size, and computational complexity.

Compared with the improved model before and after compression, the model size is reduced from 15.2 MB to 11.2 MB, GFLOPs are reduced from 18.5 to 16.3, and the single picture detection time is reduced from 7.5 ms to 6.8 ms while maintaining nearly same AP, which shows that model compression method has better results in reducing the computational resource overhead while maintaining high accuracy. In the process of sparse training, the main information in the model is gradually gathered into some important channels, while the information in the unimportant channels has little impact on the output of the results; therefore, when we remove the unimportant channels, on the one hand, we obtain a more compact model than before; on the other hand, the accuracy of the model does not have a greater impact. However, this method also has limitations. According to the theory of information entropy, there is a limit to the compression of any piece of information. Therefore, if you want to compress the model while maintaining the original accuracy, there is also a limit, rather than infinite compression.

In addition, we trained YOLOv7 (the latest algorithm of the YOLO family) [29] and FCOS with ResNet50 (an anchor free object detection method) [30] on the VisDrone dataset for 200 epochs and compared them with our method, and the results are shown in Table 3. YOLOv7 and FCOS are higher than our method in Precision, but lower in Recall and AP, and the model sizes of YOLOv7 and FCOS are generally large and not suitable for deployment on low computing power platforms. It can be seen that our method can achieve a better balance between accuracy and efficiency and is suitable for deployment on embedded devices.

**Figure 10:**
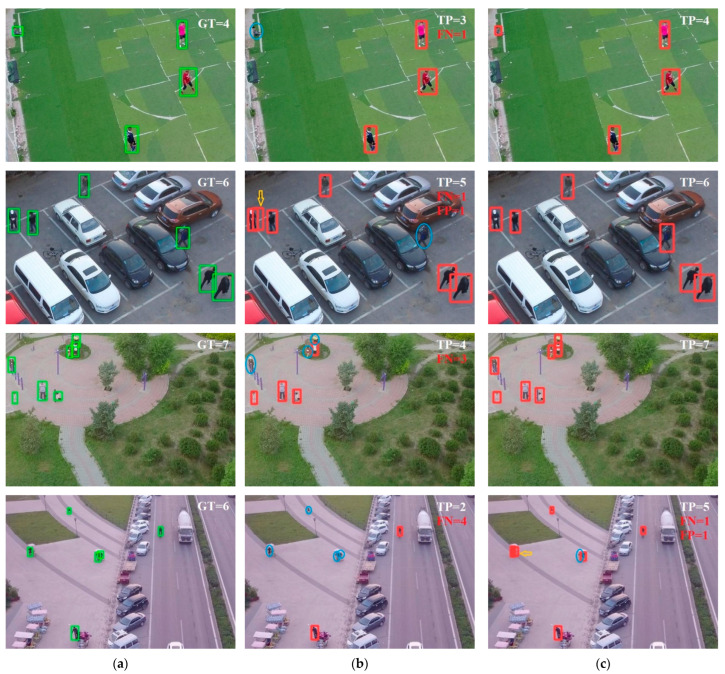
The detection results of different methods: (**a**) ground truth; (**b**) original YOLOv5s; (**c**) YOLOv5s_P2+ compression. The ground truths are marked by green boxes. Prediction results are marked by red boxes. The missed detections are marked by blue ellipses. The false detections are marked by yellow arrows. GT means the number of ground truths. TP means the number of right detections. FN means the number of missed detections. FP means the number of false detections.

Figure 10 shows the comparison of the detection results of the original YOLOv5s model and the compressed YOLOv5s_P2. The first column shows the ground truths of dataset, the second column shows the detection results of the YOLOv5s model, and the third column shows the detection results of the compressed YOLOv5s_P2.

In general, the problem of small target detection was solved to a certain extent. Taking the first line and the last line of images as an example: the person sitting on the ground in the upper left corner of the first line of images and the people riding on the square in the upper left corner of the fourth line of pictures were detected in the compressed YOLOv5s_P2, which is too small for YOLOv5 to detect.

In addition, the introduction of the small object detection layer also promoted the detection of the other three detection layers, integrating more location information and semantic information to make object detection more accurate. Taking the second line and the third line of images as an example: in the second line, the target marked by blue ellipse is easily confused with the background, which leads to missed detection in the YOLOv5s model; in the third line, the three people gathered at the top of the image are close to each other, which leads to occlusion phenomenon and the YOLOv5s model only detected one of the three people. However, in the results of compressed YOLOv5s_P2, these two problems did not occur.

But compressed YOLOv5s_P2 has some problems. For example, in the last line of images, compressed YOLOv5s_P2 obtained one missed detection and one false detection, which shows that the method in this paper has limitations.

## 5. Conclusions

The task of pedestrian target detection in UAV images is one of the research hotspots in the field of remote sensing. In this paper, a small object detection layer is introduced to YOLOv5, and after sparse training and channel pruning, high accuracy and recognition rates are achieved on the research dataset. The method has better performance and higher efficiency compared with YOLOv5, and is one of the effective solutions for pedestrian target detection in UAV images. In the subsequent research, we will continue to optimize the model and improve the detection speed of the model to achieve real-time pedestrian detection for UAV embedded platforms.

## Figures and Tables

**Table 1:** Anchors obtained by clustering on the VisDrone dataset.

Model	Detection Heads	Anchors
YOLOv5s	P3	[3, 6], [4, 10], [7, 9]
	P4	[6, 13], [9, 13], [7, 18]
	P5	[10, 22], [16, 21], [18, 36]
YOLOv5s_P2	P2	[3, 5], [4, 8], [6, 8]
	P3	[4, 12], [6, 13], [9, 12]
	P4	[7, 17], [12, 15], [10, 23]
	P5	[14, 26], [27, 27], [23, 50]

**Table 2:** Experimental results of our methods.

Model	Precision Rate	Recall Rate	AP	Model Size (MB)	Single Picture Detection Time (ms)	GFLOPs
YOLOv5s	0.701	0.507	0.572	14.4	4.6	15.8
YOLOv5s_P2	0.714	0.541	0.616	15.2	7.5	18.5
YOLOv5s_P2+ Compression	0.733	0.542	0.612	11.2	6.8	16.3

**Table 3:** Experimental results of different models.

Model	Precision Rate	Recall Rate	AP	Model Size (MB)
YOLOv5s_P2+ Compression	0.733	0.542	0.612	11.2
YOLOv7	0.902	0.282	0.525	149.2
FCOS	0.856	0.274	0.463	128.8

## Data Availability

Not applicable.

## Abbreviations

The following abbreviations are used in this manuscript:

UAV	Unmanned Aerial Vehicle
YOLO	You Only Look Once
RCNN	Region Convolutional Neural Network
RPN	Region Proposal Network
SPP	Spatial Pyramid Pooling
FPN	Feature Pyramid Network
PAN	Path Aggregation Network
BN	Batch Normalization
AP	Average Precision
TP	True Positive
FP	False Positive
FN	False Negative
IoU	Intersection over Union
